# Studies in humanized mice and convalescent humans yield a SARS-CoV-2 antibody cocktail

**DOI:** 10.1126/science.abd0827

**Published:** 2020-06-15

**Authors:** Johanna Hansen, Alina Baum, Kristen E. Pascal, Vincenzo Russo, Stephanie Giordano, Elzbieta Wloga, Benjamin O. Fulton, Ying Yan, Katrina Koon, Krunal Patel, Kyung Min Chung, Aynur Hermann, Erica Ullman, Jonathan Cruz, Ashique Rafique, Tammy Huang, Jeanette Fairhurst, Christen Libertiny, Marine Malbec, Wen-yi Lee, Richard Welsh, Glen Farr, Seth Pennington, Dipali Deshpande, Jemmie Cheng, Anke Watty, Pascal Bouffard, Robert Babb, Natasha Levenkova, Calvin Chen, Bojie Zhang, Annabel Romero Hernandez, Kei Saotome, Yi Zhou, Matthew Franklin, Sumathi Sivapalasingam, David Chien Lye, Stuart Weston, James Logue, Robert Haupt, Matthew Frieman, Gang Chen, William Olson, Andrew J. Murphy, Neil Stahl, George D. Yancopoulos, Christos A. Kyratsous

**Affiliations:** 1 Regeneron Pharmaceuticals, Inc., Tarrytown, NY 10591, USA.; 2 National Centre for Infectious Diseases, Tan Tock Seng Hospital, Yong Loo Lin School of Medicine, Lee Kong Chian School of Medicine, 16 Jalan Tan Tock Seng, Singapore 308442, Singapore.; 3 Department of Microbiology and Immunology, University of Maryland School of Medicine, Baltimore, MD 21201, USA.

## Abstract

Neutralizing antibodies have become an important tool in treating infectious diseases. Recently, two separate approaches yielded successful antibody treatments for Ebola—one from genetically humanized mice and the other from a human survivor. Here, we describe parallel efforts using both humanized mice and convalescent patients to generate antibodies against the severe acute respiratory syndrome coronavirus 2 (SARS-CoV-2) spike protein, which yielded a large collection of fully human antibodies that were characterized for binding, neutralization, and three-dimensional structure. On the basis of these criteria, we selected pairs of highly potent individual antibodies that simultaneously bind the receptor binding domain of the spike protein, thereby providing ideal partners for a therapeutic antibody cocktail that aims to decrease the potential for virus escape mutants that might arise in response to selective pressure from a single-antibody treatment.

## An antibody cocktail against SARS-CoV-2

There is an urgent focus on antibodies that target the severe acute respiratory syndrome coronavirus 2 (SARS-CoV-2) viral spike and prevent the virus from entering host cells. Hansen *et al.* generated a large panel of antibodies against the spike protein from humanized mice and recovered patients. From this panel, they identified several neutralizing antibodies, including pairs that do not compete for binding to the receptor binding domain. Baum *et al.* focused in on four of these antibodies. All four are effective against known spike variants. However, by growing a pseudovirus that expresses the spike in the presence of individual antibodies, the authors were able to select for spike mutants resistant to that antibody. In contrast, escape mutants are not selected when pseudovirus is grown in the presence of pairs of antibodies that either do not compete or only partially compete for binding to the RBD. Such a pair might be used in a therapeutic antibody cocktail.

*Science*, this issue p. 1010, p. 1014

## Teaser

A noncompeting antibody cocktail to SARS-CoV-2 spike protein safeguards against the ability of virus mutations to lead to antibody resistance.

In the setting of the current coronavirus disease 2019 (COVID-19) pandemic, there has been urgency to develop potent antiviral treatments, and early efforts have hearkened back to the days of Emil von Behring, who won the Nobel prize for showing that antibodies can be transferred in serum. However, technological advances over the last century have allowed for the progression from using convalescent serum to the utilization of recombinant fully human antibodies. The proposal to genetically humanize the immune system of mice (*1*) has provided an efficient source of naturally selected, fully human antibodies. For example, such mice have been used to develop checkpoint inhibitors for immune oncology (*2*) as well as Food and Drug Administration (FDA)–approved antibodies for the treatment of rheumatoid arthritis, cardiovascular disease, cutaneous squamous cell carcinoma, and allergic diseases such as asthma and atopic dermatitis. More recently, the ability to sort individual B cells from previously infected human patients and molecularly clone the antibody genes from these B cells has led to an independent source of human antibodies, albeit limited to antibodies that target infectious agents. Recently, these two fundamentally different approaches were independently exploited to develop fully human antibody treatments for the lethal infectious disease caused by the Ebola virus: Genetically humanized VelocImmune (VI) mice (*3*, *4*) generated an Ebola antibody cocktail treatment (*5*), whereas sorting B cells from a recovered patient yielded a single–therapeutic antibody treatment (*6*).

In this work, we describe parallel high-throughput efforts using both mice and humans to generate antibodies against the spike protein of severe acute respiratory syndrome coronavirus 2 (SARS-CoV-2). The ability to derive antibodies using genetically humanized VI mice as well as B cells derived from convalescent patients enabled us to isolate a very large collection of fully human antibodies with diverse sequences, binding properties, and antiviral activities. The prospective goal of these parallel efforts was to generate a large and diverse collection so as to allow for the selection of pairs of highly potent individual antibodies that could simultaneously bind the critical receptor binding domain (RBD) of the spike protein, thereby providing ideal partners for a therapeutic antibody cocktail that would have the potential to decrease the likelihood of virus escape mutants that might arise in response to selective pressure from single-antibody treatments (*7*).

Anti–SARS-CoV-2 spike antibodies were generated with the following two methods. First, VI mice were immunized with a DNA plasmid that expresses SARS-CoV-2 spike protein and then were boosted with a recombinant protein composed of the RBD of SARS-CoV-2 spike. Second, antibodies were isolated from peripheral blood mononuclear cells (PBMCs) of human donors previously infected with SARS-CoV-2. VI mice elicited a robust immune response against the SARS-CoV-2 spike protein after immunization. Titers of mice blood collected 7 days after the last boost were determined by enzyme-linked immunosorbent assay (ELISA) (fig. S1). Mice with the highest titers were used for antibody isolation. Spleens from these mice were subjected to biotin-labeled monomeric RBD antigen staining and fluorescence-activated single-cell sorting. In parallel, whole blood was collected from three patients 3 to 4 weeks after a laboratory-confirmed polymerase chain reaction (PCR) positive test for SARS-CoV-2 and after showing symptoms of COVID-19. PBMCs were isolated by ficoll gradient and RBD-specific B cells were fluorescence-activated single-cell sorted. The first sets of antibodies derived from these platforms are described here.

To assess antigen-specific responses, naturally paired heavy and light chain cDNAs were cloned from the mice and human-derived B cells (*8*) and transfected into Chinese hamster ovary (CHO) cells to produce recombinant fully human antibodies. Cultured supernatants containing secreted antibodies were subjected to high-throughput screening for RBD protein binding. Thousands of antibodies were isolated and subsequently screened for binding affinity to RBD monomer and dimer, epitope diversity, ability to block angiotensin-converting enzyme 2 (ACE2) receptor binding to RBD, and ability to neutralize vesicular stomatitis virus (VSV)–based SARS-CoV-2 spike pseudoparticles [pVSV-SARS-CoV-2-S(mNeon)]. Screening yielded >200 neutralizing monoclonal antibodies (mAbs) with broad potency ranges, dozens of which displayed neutralization potency in the picomolar range.

**Fig. 1:**
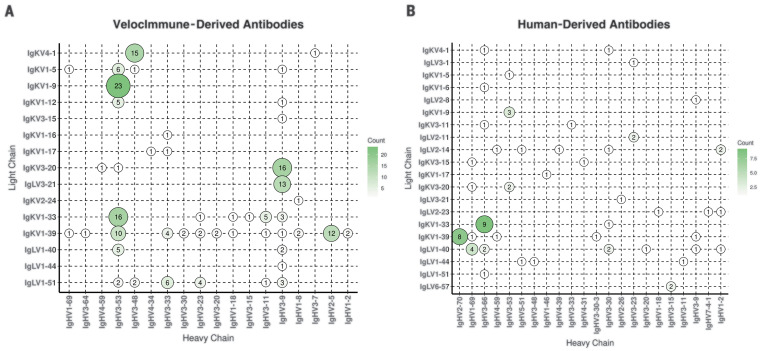
Paired antibody repertoire for human- and mouse-derived SARS-CoV-2 neutralizing antibodies. (**A** and **B**) Variable (V) gene frequencies for paired heavy (x axes) and light (y axes) chains of isolated neutralizing antibodies to SARS-CoV-2 for VI mice (A) (*N* = 185) and convalescent human donors (B) (*N* = 68). The color and size of the circles correspond to the number of heavy and light chain pairs present in the repertoires of isolated neutralizing antibodies. Neutralization is defined as >70% with 1:4 dilution of antibody (~2 μg/ml) in VSV-based pseudoparticle neutralization assay.

More than 200 of the VI mouse and human-derived antibodies isolated in the primary screen neutralized VSV-based SARS-CoV-2 spike pseudoparticles at >70% with ~2 μg/ml of expressed antibodies. The antibody variable regions were sequenced by next-generation sequencing, and the repertoire for heavy and light chain pairs was identified (Fig. 1). The predominant lineage of VI mouse antibodies utilized VH3-53 paired with VK1-9, VK1-33, or VK1-39, whereas our human-derived antibodies utilized VH3-66 paired with VK1-33 or VH2-70 paired with VK1-39. Notably, VH3-53 usage has recently been reported for another human-derived potent neutralizing antibody against SARS-CoV-2 spike protein (*9*–*11*), which indicates that combining the VI mouse approach with the human platforms allows the expanded capture of common rearrangements found in potent neutralizing SARS-CoV-2 mAbs seen in humans. Further analysis of overlaid sequences (fig. S2) showed strong overlap in the repertoire of isolated kappa chains between VI mouse and human-derived antibodies. Although the repertoire of lambda chains did not overlap well, that may be because only two lambda mice were included in this trial. The average complementarity-determining region (CDR) lengths (fig. S2D) for heavy chains was similar between VI mouse and human-derived antibodies, with average lengths of 13 and 14.5 amino acids, respectively. The average kappa CDR length (fig. S2E) was the same for VI mouse and human-derived antibodies at 9 amino acids, and the lengths were similar for lambda chains (fig. S2F), with an average length of 11.1 and 10.6 amino acids, respectively.

**Fig. 2:**
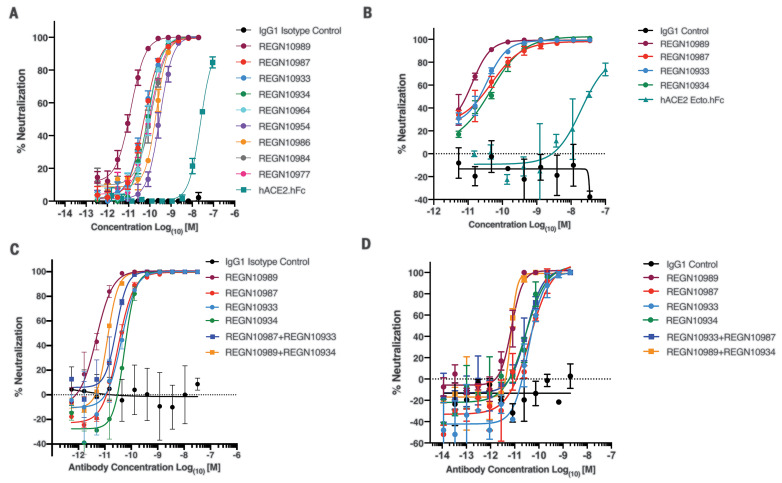
Neutralization potency of anti–SARS-CoV-2 spike mAbs. (**A**) Serial dilutions of anti-spike mAbs, IgG1 isotype control, and recombinant dimeric ACE2 (hACE2.hFc) were added with pVSV-SARS-CoV-2-S(mNeon) to Vero cells, and mNeon expression was measured 24 hours after infection as a readout for virus infectivity. Data are graphed as percent neutralization relative to virus-only infection control. (**B**) Neutralization potency of anti-spike mAbs, recombinant dimeric ACE2, and IgG1 isotype control against nonreplicating pVSV-SARS-CoV-2-S(mNeon) in Calu-3 cells. (**C**) Neutralization potency of individual anti-spike mAbs and combinations of mAbs against replicating VSV-SARS-CoV-2-S virus in Vero cells. Cells were infected with a multiplicity of infection (MOI) 1 of the virus and stained for viral protein 24 hours after infection to measure infectivity. (**D**) Neutralization potency of individual anti-spike mAbs and combinations of mAbs against SARS-CoV-2-S virus in VeroE6 cells.

Approximately 40 antibodies with distinct sequences and potent neutralization activities were chosen for additional characterization, as described below. The neutralization potency of these mAbs spanned the single-digit to triple-digit picomolar range in the VSV-based pseudoparticle assay. Antibodies shown to cross-neutralize SARS-CoV-1 and SARS-CoV-2 spike proteins were weakly neutralizing (*12*). So instead of focusing on cross-neutralizers, we focused on nine of the most potent neutralizing mAbs, with neutralization potencies ranging from 7 to 99 pM (Fig. 2A and table S1). All of these neutralizing mAbs bound to the RBD of SARS-CoV-2 spike and blocked its ability to interact with ACE2 with double-digit picomolar median inhibitory concentrations (IC_50_s) (table S1), which supports ACE2 blockade as the primary mechanism for neutralization. The antibodies bound specifically and with high affinity to monomeric SARS-COV-2 RBD [dissociation constant (*K*_d_) = 0.56 to 45.2 nM] and dimeric SARS-COV-2 RBD (*K*_d_ = 5.7 to 42.8 pM). Because recombinant ACE2 receptor is being considered as a COVID-19 therapeutic (*13*), we tested the potency of recombinant dimeric human ACE2-Fc (hACE2-hFc) in our neutralization assay. Although recombinant ACE2 was able to mediate neutralization of the VSV-based spike pseudoparticles as previously reported, its potency was reduced by more than a factor of 1000 compared with that of the best neutralizing mAbs (Fig. 2, A and B).

A smaller collection of four antibodies was chosen for further analyses to determine whether the above binding data to RBD reflected binding to trimeric spike protein, whether neutralization potencies noted in the above assays were consistent with those seen in other assays including with SARS-CoV-2, and whether these antibodies retained neutralization activity against pseudoparticles with mutations in the S1-S2 cleavage site. The binding affinity of these four antibodies against trimeric SARS-CoV-2 spike (*K*_d_ = 37.1 to 42.8 pM) largely paralleled the affinity against the RBD (table S3). Additionally, the potent neutralizing activity of these four antibodies was confirmed in the additional neutralization assays, including neutralization of pVSV-SARS-CoV-2-S(mNeon) in the human lung epithelial Calu-3 cell line, neutralization of replicating VSV-SARS-CoV-2-S in Vero cells, and neutralization of SARS-CoV-2 in VeroE6 cells (Fig. 2, B to D). All neutralization assays generated similar potency across the four mAbs, and no combinations demonstrated synergistic neutralization activity (Fig. 2, C and D). As previous studies indicate pseudoparticles containing the SARS-CoV-2 spike are precleaved by furin-like proteases at the polybasic S1-S2 cleavage site during biogenesis in HEK293T cells, we assessed the impact of this cleavage on mAb neutralization potency. Spike-stabilized pseudoparticles (fig. S3A) with a monobasic cleavage site (FurMut) in the S1-S2 interface or deleted region (FurKO) were produced as previously described (*14*, *15*). No differences were observed in neutralization of either FurMut- or FurKO-containing pseudoparticles relative to wild-type (WT) in Vero cells (fig. S3B). Notably, stabilized pseudoparticles had comparable or greater infectivity to those with WT cleavage sites in Vero cells, whereas substantial loss of infectivity was observed in Calu-3 cells (fig. S3C). Authentic SARS-CoV-2 with a natural deletion of the S1-S2 junction also had defects in infectivity in Calu-3 but not in Vero cells (*16*), which implicates differential protease usage between these two cell types. To investigate the mechanism of neutralization, we generated antigen-binding fragments (Fabs) for the four antibodies. We compared immunoglobulin G (IgG) with corresponding Fabs side by side for their ability to neutralize pseudotyped VSV (fig. S4). The IC_50_s of all the Fabs were shifted compared with those of their parental IgGs, which indicates that bivalent binding, cross-linking, and steric hindrance might all augment neutralization.

Although the role of antibody effector function in protection against SARS-CoV-2 is yet unknown, it has been well established that it plays an important role in mAb therapeutic efficacy against other viruses such as Ebola and influenza viruses (*17*–*19*). Effector cells including macrophages and monocytes have also been shown to be important for antibody-mediated protection from SARS-CoV-1 infection (*20*). To understand whether our lead antibodies are capable of mediating effector function, we assessed both antibody-dependent cellular cytotoxicity (ADCC) and antibody-dependent cellular phagocytosis (ADCP) activity in primary human cell bioassays utilizing natural killer (NK) cells and monocyte-derived phagocytes. All four lead antibodies demonstrated the ability to mediate ADCC and ADCP, albeit to slightly different degrees. REGN10987 displayed superior ability to mediate ADCC relative to the other three mAbs, whereas it performed similarly to REGN10989 and REGN10933 in the ADCP assay (fig. S5 and table S3). Although REGN10934 was able to mediate both ADCC and ADCP, it was not as strong of an inducer in those assays as the other three mAbs (fig. S6 and table S4). Further identification of mAb epitopes through high-resolution structural analysis may help illuminate the relationship between specific epitopes and effector function of anti-spike mAbs.

**Fig. 3:**
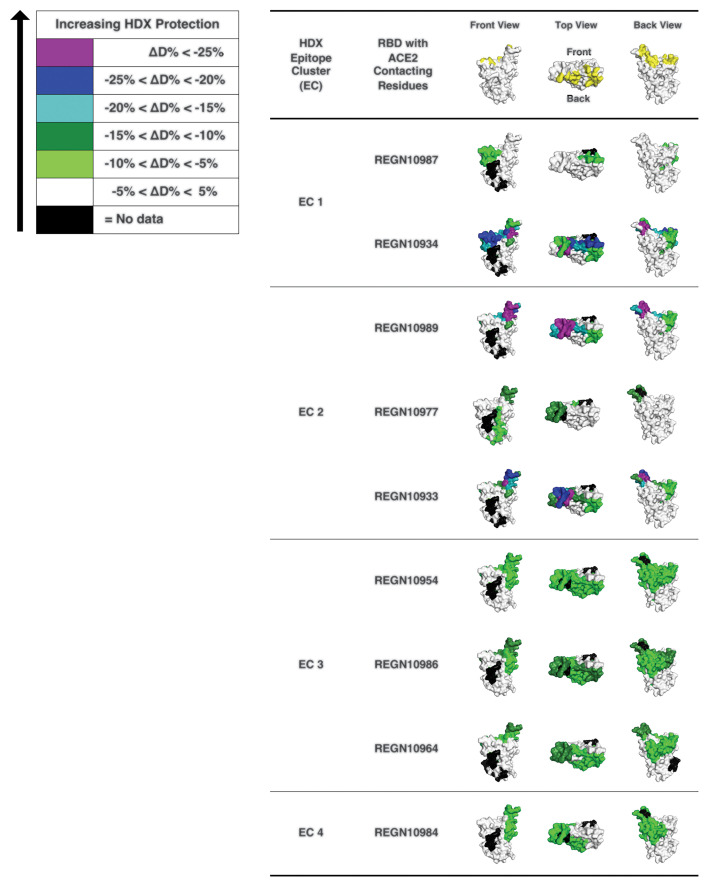
HDX-MS determines mAb interaction on spike protein RBD. 3D surface models for the structure of the spike protein RBD domain showing the ACE2 interface and HDX-MS epitope mapping results. RBD residues that make contacts with ACE2 (*21*, *22*) are indicated in yellow (top). RBD residues protected by anti–SARS-CoV2 spike antibodies are indicated with colors that represent the extent of protection, as determined by HDX-MS experiments. RBD residues in purple and blue indicate sites of lesser solvent exchange upon antibody binding that have greater likelihood to be antibody-binding residues. The RBD structure is reproduced from PDB 6M17 (*21*).

**Fig. 4:**
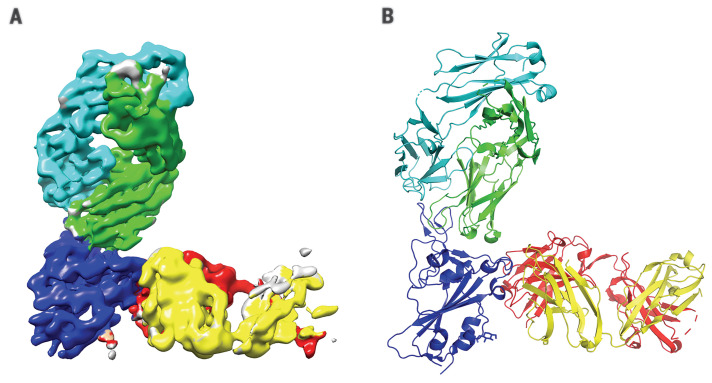
Complex of REGN10933 and REGN10987 with the SARS-CoV-2 RBD. (**A**) 3.9-Å cryo-EM map of the REGN10933-RBD-REGN10987 complex, colored according to the chains in the refined model (**B**). RBD is colored dark blue; REGN10933 heavy and light chains are green and cyan, respectively; and REGN10987 heavy and light chains are yellow and red, respectively.

A prospective goal of our effort was to identify highly potent individual antibodies that could be paired in a therapeutic antibody cocktail, aiming to decrease the potential for decreased efficacy caused by variants arising as the pandemic spreads or by virus escape mutants that might be selected for in response to pressure from a single-antibody treatment (*7*). Thus, we examined our nine most-potent neutralizing antibodies in cross-competition binding assays (fig. S7) and identified several pairs of noncompeting mAbs with picomolar neutralization potency that could potentially be combined to form antibody cocktails. To further study the binding regions of our mAbs on spike protein RBD, we performed hydrogen-deuterium exchange mass spectrometry (HDX-MS) with the same nine antibodies (Fig. 3), which revealed where each of the antibodies contacts the surface of the RBD and allowed comparison with the ACE2 binding site on the RBD (Fig. 3). As might be expected, most of our neutralizing antibodies contact the RBD in a manner that overlaps the RBD residues that comprise the ACE2 interface; furthermore, the antibodies can be grouped on the basis of their pattern of contacting the RBD surface. Comparing the cross-competition binding assays with the HDX-MS results provides structural insights into the mechanism by which noncompeting pairs of antibodies can simultaneously bind the RBD and can thus be ideal partners for a therapeutic antibody cocktail. REGN10987 and REGN10933 represent such a pair of antibodies: REGN10933 targets the spike-like loop region on one edge of the ACE2 interface. Within that region, the residues that show the most notable HDX protection by REGN10933 face upward, which suggests that the Fab region of REGN10933 binds the RBD from the top direction, where REGN10933 will have collisions with ACE2. To avoid competition with REGN10933, REGN10987 can only bind to the HDX-defined protected regions from the front or the lower left side (in the front view of REGN10987 in Fig. 3). This would be consistent with the neutralization data, as REGN10987 would orient itself in a position that has high probability to interfere with ACE2. Confirming the above data, single-particle cryo–electron microscopy (cryo-EM) of the complex of SARS-CoV-2 spike RBD bound to Fab fragments of REGN10933 and REGN10987 shows that the two antibodies in this cocktail can simultaneously bind to distinct regions of the RBD (Fig. 4 and table S5). A three-dimensional (3D) reconstructed map of the complex with nominal resolution of 3.9 Å shows that the two Fab fragments bind at different epitopes on the RBD, which confirms that they are noncompeting antibodies. REGN10933 binds at the top of the RBD, extensively overlapping the binding site for ACE2. On the other hand, the epitope for REGN10987 is located on the side of the RBD, away from the REGN10933 epitope, and has little to no overlap with the ACE2 binding site.

We report notable similarities and consistencies in the antibodies generated from genetically humanized mice and from convalescent humans. The scale of the genetic-engineering approach used to create the VI mouse (involving genetic-humanization of more than 6 Mb of mouse immune genes) has resulted in the ability to effectively and indistinguishably mimic the antibody responses of normal humans. The genetically humanized–mouse approach has the advantages that it can potentially allow for further immunization optimization strategies and that it can be applied to noninfectious disease targets. By combining the efforts from two parallel and high-throughput approaches for generating antibodies to the RBD of the SARS-CoV-2 spike protein, we generated a sufficiently large collection of potent and diverse antibodies that we could meet our prospective goal of identifying highly potent individual antibodies that could be combined into a therapeutic antibody cocktail. Inclusion of such antibodies into an antibody cocktail may deliver optimal antiviral potency while minimizing the odds of virus escape (*7*)—two critical, desired features of an antibody-based therapeutic for treatment and prevention of COVID-19. Such an antibody cocktail is now being tested in human trials (clinicaltrials.gov NCT04426695 and NCT04425629).

## Supplementary Materials

science.sciencemag.org/content/369/6506/1010/suppl/DC1 Materials and Methods Figs. S1 to S7 Tables S1 to S5 References (*23*–*33*) MDAR Reproducibility Checklist Data S1

## Appendix

View/request a protocol for this paper from *Bio-protocol*.
